# High variability in what is considered important to report following instability surgery: a Delphi study among Dutch shoulder specialists

**DOI:** 10.1016/j.jseint.2023.06.020

**Published:** 2023-07-23

**Authors:** Lukas P.E. Verweij, Just A. van der Linde, Derek F.P. van Deurzen, Michel P.J. van den Bekerom, the SINC Study Group, E.E.J. Raven, M.P.J. van den Borne, O.A.J. van der Meijden, T.D.W. Alta, R.N. Wessel, A. van Noort, T. Gosens, Y.V. Kleinlugtenbelt, T.D. Berendes, H.C. van der Veen, C. Visser, O.F.O. Lambers Heerspink, O. van der Meer, I. Bonneux, S. Floor, D.P. van Oostveen

**Affiliations:** a Amsterdam UMC, Location AMC, University of Amsterdam, Department of Orthopedic Surgery and Sports Medicine, Amsterdam, the Netherlands; b Amsterdam Movement Sciences, Musculoskeletal Health Program, Amsterdam, the Netherlands; c Amsterdam Shoulder and Elbow Center of Expertise (ASECE), Amsterdam, the Netherlands; d Reiner Haga Orthopaedic Centre, Zoetermeer, the Netherlands; e Shoulder and Elbow Unit, Joint Research, Department of Orthopedic Surgery, OLVG, Amsterdam, the Netherlands; f Department of Orthopedic Surgery, Medical Center Jan van Goyen, Amsterdam, the Netherlands; g Faculty of Behavioural and Movement Sciences, Department of Human Movement Sciences, Vrije Universiteit Amsterdam, Amsterdam, the Netherlands

**Keywords:** Delphi, Consensus, Standardized Reporting, Shoulder, Instability, Surgical report, Surgery

## Abstract

**Background:**

Standardized reporting leads to high-quality data and can reduce administration time. The aim of this study was to (1) get an insight into the variability of what is considered important to report in the surgical report following shoulder instability surgery and (2) determine which elements should be included in the surgical report following shoulder instability surgery according to Dutch surgeons using a Delphi method.

**Methods:**

Dutch orthopedic shoulder surgeons were included in a panel for a Delphi study consisting of 3 rounds. Importance of the elements was rated on a 9-point Likert scale. High variability was defined as an element that received at least 1 score between 1 and 3 and 1 score between 7 and 9 in round 3. Consensus was defined as ≥80% of the panel giving a score of 7 or more.

**Results:**

Seventeen shoulder specialists completed all 3 rounds and identified a total of 82 elements for the arthroscopic Bankart repair and 60 for the open Latarjet. High variability was observed in 57 (70%) and 52 (87%) of the elements, respectively. After round 3, the panel reached consensus on 27 and 11 elements that should be mentioned in the surgical report following arthroscopic Bankart repair and open Latarjet.

**Conclusion:**

There is high variability in what shoulder specialists regard essential to report. Consensus was reached on 27 and 11 elements to be reported following arthroscopic Bankart repair and open Latarjet, respectively. Future studies on an international scale can further improve data collection and communication between specialists.

Many shoulder instability publications are characterized by heterogeneous patient populations, different patient or pathological factors leading to surgery, and heterogeneous outcome parameters. To date, studies that aim to identify risk factors for recurrent instability following surgical treatment have shown conflicting results leading to high heterogeneity in meta-analyses.^14^^,^^21^ Although several studies focus on consensus regarding treatment strategies for patients with clear cut indications, such as (large) bone defects or high shoulder demand,^7^^,^^9^^,^^11^, ^12^, ^13^^,^^16^^,^^19^ there is still a large grey area where it is unclear which patients should receive which treatment because the best available evidence for risk-stratification is based on heterogeneous outcomes.^20^

Standardization of data collection and outcomes parameters will likely optimize both the production and quality of clinical research, as a similar format will facilitate data sharing and clear definitions prevent bias when data are combined in a database.^2^^,^^22^ During training, residents are taught by their mentors how to perform surgery and what information to report in the surgical file. However, there has never been a consensus study that investigates which peroperative elements are deemed important to report by shoulder specialists. In recent years, more studies have focused on combining retrospective data in databases that can be used to identify factors that predict a specific outcome used in clinical prediction models or for stratification in trials.^18^ It is crucial to start thinking about *what* data need to be collected to identify risk factors and *how* we want to collect these data to prevent bias and missing data in the databases that are used for clinical research.^15^^,^^17^^,^^23^ Even the definition of the most commonly used outcome in shoulder instability, recurrence, demonstrates high variability in clinical studies.^1^ This demonstrates the need for standardized measuring and reporting to prevent bias when comparing or combining study data. Besides from a research point of view, standardized reporting can also make it more clear for other surgeons how a procedure was performed when they take over a case or when a case needs to be assessed for medical legal reasons.

Reaching a consensus on what to report in the surgical report can be a challenging process. Even more so, implementing this consensus into their clinical work, as surgeons have limited time, might be challenging as they have to change their administrative behavior and specialists are not always research-driven. In addition, administration is considered dull and the load already exceeds what most surgeons find acceptable. Therefore, it is important to create awareness of the benefits of standardized reporting and determine to what extent consensus on reporting already exists. Consensus with regard to irrelevant aspects could also decrease the administrative load as some elements that are currently reported could be omitted, which will help in further improving communication between specialists. A Delphi study is a well-accepted iterated method to assemble experts and reach a consensus for a controversial topic.^8^^,^^9^ The Delphi study design comprises multiple anonymous rounds in which the experts can share their opinion and receive feedback from other participants.^8^, ^9^, ^10^ The goal of this study was to start the conversation about data collection, data management, and definitions in shoulder instability research to improve homogeneous data collection and communication between specialists. Therefore, the aim was to (1) get an insight into the variability of what is considered important to report in the surgical report following shoulder instability surgery and (2) determine which elements should be included in the surgical report following shoulder instability surgery according to Dutch surgeons using a Delphi method.

## Methods

The recommendations for methodologic criteria and reporting for Delphi studies by Diamond et al and Hohmann et al were used.^4^^,^^8^^,^^9^ The lead author (L.P.E.V.) served as liaison and created the questionnaires based on the responses. The liaison handled all communication and did not participate in the study to prevent bias in the analyses. All questionnaires were sent using Castor EDC (Amsterdam, the Netherlands), which is an online tool to send questionnaires and collect data. The Delphi study consisted of 3 rounds, comprising (1) identifying elements that need to be reported according to the shoulder specialists, (2) rating the importance of these elements, and (3) rating the importance again after seeing a summary of the results with feedback of the participants of round 2. The surgical report for the arthroscopic Bankart repair and open Latarjet was evaluated, as these procedures are most commonly performed by shoulder specialists in the Netherlands.

### Assembling the expert panel

Shoulder specialists who perform surgical treatment to treat anterior shoulder instability were asked to participate. The network of the authors was used to find suitable candidates for participating in the Delphi study, whom all recruited members through the Dutch Shoulder and Elbow Society (WSE). A consensus on how many experts should be included in a Delphi study is yet to be reached, as this could vary based on the topic, but a previous study by Hsu et al advised to include 10 to 15 subjects if their background is homogenous.^10^ Therefore, the aim was to include at least 15 shoulder specialists in the panel.

### First round

The first round identified elements that need to be reported according to the shoulder surgeons through 2 open-ended questions. These questions included *“Which elements do you believe should always be included in the surgical report following an arthroscopic Bankart repair?”* and *“Which elements do you believe should always be included in the surgical report following an open Latarjet procedure?”* To make the list of elements more accessible, they were categorized in domains. These domains were discussed and checked with the study team for accuracy. If disagreement arose regarding any of the descriptions, the study team discussed a more appropriate use of words. For the arthroscopic Bankart repair, the domains included *“standard and patient factors”, “preoperative tests”, “labrum”, “lesions”, “surgery specific/technical elements”, “fixation”, “additional surgical interventions”, and “rehabilitation”*. For the open Latarjet, *“standard and patient factors”, “preoperative tests”, “graft”, “lesions”, “surgery specific/technical elements”, “fixation” , and “rehabilitation”*. This resulted in a final list of elements that could be evaluated in round 2 and 3. Furthermore, baseline characteristics regarding experience and surgical volume of the shoulder specialists were acquired.

### Second and third round

During the second round, the panel was asked to rate the importance of the elements that resulted from the first round using a 9-point Likert scale (1 being not important and 9 being very important) according to the questions *“How important do you consider this element to always be reported in the surgical report following an arthroscopic Bankart repair?”* and *“How important do you consider this element to always be reported in the surgical report following an open Latarjet procedure?”*.^6^^,^^9^ The results of round 2 were summarized in histograms that showed how many participants rated the elements with a score of 7 or more. This summary was presented to the panel before they entered round 3. After seeing the summary of round 2, the panel rated the elements again in round 3. Consensus was defined as ≥80% of the panel giving a score between either 1 and 3 (element not important to report) or 7 and 9 (element should always be reported) in round 3. When ≥90% of the panel gave these scores, it was defined as a strong consensus.

### Data collection and analysis

Baseline demographic data were presented as average and standard deviation or median and interquartile range according to their distribution. These included age, sex, years of experience, and surgical volume. High variability was defined as an element that received at least 1 score between 1 and 3 and 1 score between 7 and 9 in round 3. The categorical variable ‘consensus’ was expressed as absolute numbers (percentages). Partial deletion was applied to account for missing data if it was present. Data were analyzed using Excel (Microsoft Excel 2018; Microsoft Corp., Redmond, WA, USA).

## Results

### Panel characteristics and identified elements

A total of 22 shoulder specialists were contacted by e-mail and 19 (86%) agreed to participate in this Delphi study between June 1 and October 31, 2022. All 3 rounds were completed by 17 (89%) shoulder specialists (Table I). After round 1 was completed, a total of 82 different elements were identified for the arthroscopic Bankart repair and 60 different elements were identified for the open Latarjet procedure.

**Table I:** Characteristics of the panel.

Healthcare providers (n = 17)	
Age, *average* (SD)	46.9 (5.8)
Male sex, n (%)	15 (88)
Years of experience, median (IQR)	11 (7)
Years of experience as shoulder specialist, median (IQR)	9 (8)
Annual volume, median (IQR)	
Arthroscopic Bankart repair	20 (10)
Open Latarjet	10 (5)

*SD*, standard deviation; *IQR*, interquartile range.

### Third round: arthroscopic Bankart repair

After seeing the summary of round 2, the panel reached consensus on 27 elements for the arthrosopic Bankart repair, of which 12 reached a strong consensus (Table II; Supplements 1). These elements included side (88%), surgical indication (88%), if the labrum could reach the glenoid rim (94%), if there was a normal variation of the labrum (94%), location of the labral lesion (94%) according to the clock method (88%), size of the labral lesion according to the clock method (82%), quality/integrity of the labrum at the lesion (82%), aspect of the humeral (100%) and glenoid (100%) cartilage and grading of any cartilage lesions (88%), presence of a rotator cuff lesion (94%), presence of biceps pathology (94%), integrity of the biceps anchor (94%), description of the degree of glenoid erosion (88%), presence of a Hill-Sachs lesion (88%), whether there were any events during the operation that deserve special notice (100%), whether a biceps tenotomy/tenodesis was performed (88%), whether a capsular shift was performed (88%), how many anchors were used for fixation (94%) and the position of these anchors according to the clock method (82%), whether a Hill-Sachs remplissage was performed (100%), whether a rotator cuff was repaired if a tear was present (94%), whether a bony Bankart fragment was fixated with a screw if it was present (88%), whether a humeral avulsion glenohumeral ligament repair was performed if this lesion was present (82%), duration of immobilization (88%), and physiotherapy instructions (82%; Supplements 1). None of the elements reached a consensus to definitely not be reported. High variability was observed in 57 (70%) elements.

**Table II:** Elements for arthroscopic Bankart repair report.

Strong consensus (≥90%)	Consensus (≥80%)
If the labrum could reach the glenoid rim	Side
If there was a normal variation of the labrum	Surgical indication
Location of the labral lesion	Location of the labral lesion according to the clock method
Aspect of the humeral cartilage	Size of the labral lesion according to the clock method
Aspect of the glenoid cartilage	Quality/integrity of the labrum at the lesion
Presence of a rotator cuff lesion	Grading of any cartilage lesions
Presence of biceps pathology	Description of the degree of glenoid erosion
Integrity of the biceps anchor	Presence of a Hill-Sachs lesion
Whether there were any events during the operation that deserve special notice	Whether a biceps tenotomy/tenodesis was performed
How many anchors were used for fixation	Whether a capsular shift was performed
Whether a Hill-Sachs remplissage was performed	Position of the anchors according to the clock method
Whether a rotator cuff was repaired if a tear was present	Whether a bony Bankart fragment was fixated with a screw if it was present
	Whether a HAGL repair was performed if this lesion was present
	Duration of immobilization
	Physiotherapy instructions

*HAGL*, humeral avulsion glenohumeral ligament.

### Third round: open Latarjet procedure

After seeing the summary of round 2, the panel reached consensus on 11 elements for the (mini) open Latarjet procedure, of which 4 reached a strong consensus (Table III; Supplements 1). These elements included side (94%), surgical indication (94%), whether antibiotic prophylaxis has been administered (82%), which type of graft was used (94%), a description of the position of the graft (82%) and if it was fixated flush (88%), which material was used for fixation (94%) and whether a wedge plate was used (82%), a description of the approach (88%), physiotherapy instructions (88%), and duration of immobilization (88%; Supplements 1). None of the elements reached a consensus to definitely not be reported. High variability was observed in 52 (87%) elements.

**Table III:** Elements for open Latarjet report.

Strong consensus (≥90%)	Consensus (≥80%)
Side	Whether antibiotic prophylaxis has been administered
Surgical indication	Description of the position of the graft
Type of graft	If the graft was fixated flush
Which material was used for fixation	Whether a wedge plate was used
	Description of the approach
	Physiotherapy instructions
	Duration of immobilization

## Discussion

This is the first study that evaluates consensus on reporting following an instability surgery. There was high variability observed for which elements were considered important by shoulder specialists; however, a consensus on 27 elements to be reported following an arthroscopic Bankart repair and 11 elements to be reported following an open Latarjet procedure was reached.

There are several limitations to this study and it should therefore be interpreted in the light of the following remarks: First, a part of the variability or consensus might be explained due to the fact that some shoulder specialists do not report specific elements in the surgical report but report them somewhere else as this seems more fitting to them, such as the outpatient clinic report. It was not possible to account for these differences, but it is important to take into account. Second, there is no consensus on the best design for a Delphi study. More rounds with explanation from the panel could increase the consensus and the study can be combined with an open discussion of the results. Nevertheless, the recommendations for methodologic criteria and reporting for Delphi studies by Diamond et al and Hohmann et al were used to facilitate proper design and reporting.^4^^,^^8^^,^^9^ Third, the study was performed with Dutch shoulder specialists, which might not be a representation of the international opinion. Nevertheless, the surgeons informed of the latest developments in shoulder instability research due to being active in international associations and reviewing committees for peer-review journals. Fourth, this study does not provide a one-size-fits-all solution for surgical reporting, but it creates the opportunity to standardize a set of factors in the surgical report. A fully standardized surgical report is not desirable as the surgeon needs to be able to report unexpected outcomes or peroperative complications.

Absence of agreement about reporting and definitions will likely lead to missing data and therefore bias when the data are pooled.^23^ Many loopholes have been found to account for missing data, of which partial deletion and imputation are probably the most well-known methods.^17^ However, imputation will never reach the same level of data quality as homogeneous collected data. As Sterne at al explain, missing data have the consequence of bias in the analyses.^17^ Missing data can be classified as missing completely at random, missing at random, and missing not at random.^17^ More systemic and less random missing data can lead to more bias in the analyses and therefore it is widely advised to avoid missing data. This is why guidelines such as Transparent reporting of a multivariable prediction model for individual prognosis or diagnosis and Strengthening the Reporting of Observational Studies in Epidemiology focus on missing data as well.^3^^,^^5^

The reason to report items in the clinical or surgical report can have both a clinical or scientific purpose. As more and more data are collected for these purposes, it is relevant to establish standardization with regard to the minimal set of data to collect. Future studies should focus on how we can improve standardized reporting, while keeping the administrative load to a minimum. As many different factors were proposed in the present study, it is probably possible to reduce administration time as well for some specialists.

## Conclusion

There is high variability in what shoulder specialists regard essential to report. Consensus was reached on 27 and 11 elements to be reported following arthroscopic Bankart repair and open Latarjet, respectively. Future studies on an international scale can further improve data collection and communication between specialists.

## Disclaimers

Funding: This study was funded by 10.13039/501100001826ZonMw, the Dutch association for research and innovation in healthcare.

Conflicts of interest: Derek F.P. van Deurzen is a paid instructor for Wright medical. However, this author, their immediate family, and any research foundation with which they are affiliated did not receive any further financial payments or other benefits from any commercial entity related to the subject of this article. A. van Noort: This author is a consultant at LIMA (education and clinical research). However, this author, their immediate family, and any research foundation with which they are affiliated did not receive any financial payments or other benefits from any commercial entity related to the subject of this article. The other authors, their immediate families, and any research foundation with which they are affiliated have not received any financial payments or other benefits from any commercial entity related to the subject of this article.

Patient consent: Obtained.

## References

[1] Alkaduhimi H., Connelly J.W., van Deurzen D.F.P., Eygendaal D., van den Bekerom M.P.J. (2021). High variability of the definition of recurrent glenohumeral instability: an analysis of the current literature by a systematic review. Arthrosc Sports Med Rehabil.

[2] Chalmers I., Glasziou P. (2009). Avoidable waste in the production and reporting of research evidence. Lancet.

[3] Collins G.S., Reitsma J.B., Altman D.G., Moons K.G. (2015). Transparent reporting of a multivariable prediction model for individual prognosis or diagnosis (TRIPOD): the TRIPOD statement. BMJ.

[4] Diamond I.R., Grant R.C., Feldman B.M., Pencharz P.B., Ling S.C., Moore A.M. (2014). Defining consensus: a systematic review recommends methodologic criteria for reporting of Delphi studies. J Clin Epidemiol.

[5] von Elm E., Altman D.G., Egger M., Pocock S.J., Gotzsche P.C., Vandenbroucke J.P. (2008). The strengthening the reporting of observational studies in epidemiology (STROBE) statement: guidelines for reporting observational studies. J Clin Epidemiol.

[6] Gerritsen A., Jacobs M., Henselmans I., van Hattum J., Efficace F., Creemers G.J. (2016). Developing a core set of patient-reported outcomes in pancreatic cancer: a Delphi survey. Eur J Cancer.

[7] Hohmann E. (2021). Editorial commentary: Delphi expert consensus clarifies evidence-based medicine for shoulder instability and bone loss. Arthroscopy.

[8] Hohmann E., Brand J.C., Rossi M.J., Lubowitz J.H. (2018). Expert opinion is necessary: Delphi panel methodology facilitates a scientific approach to consensus. Arthroscopy.

[9] Hohmann E., Cote M.P., Brand J.C. (2018). Research pearls: expert consensus based evidence using the Delphi method. Arthroscopy.

[10] Hsu C. (2007). The Delphi technique: making sense of consensus. Pract Assess Res Eval.

[11] Hurley E.T., Matache B.A., Wong I., Itoi E., Strauss E.J., Delaney R.A. (2022). Anterior shoulder instability part I-diagnosis, nonoperative management, and Bankart repair-an international consensus statement. Arthroscopy.

[12] Hurley E.T., Matache B.A., Wong I., Itoi E., Strauss E.J., Delaney R.A. (2022). Anterior shoulder instability part II-Latarjet, remplissage, and glenoid bone-grafting-an international consensus statement. Arthroscopy.

[13] Matache B.A., Hurley E.T., Wong I., Itoi E., Strauss E.J., Delaney R.A. (2022). Anterior shoulder instability part III-revision surgery, rehabilitation and return to play, and clinical follow-up-an international consensus statement. Arthroscopy.

[14] Olds M., Ellis R., Donaldson K., Parmar P., Kersten P. (2015). Risk factors which predispose first-time traumatic anterior shoulder dislocations to recurrent instability in adults: a systematic review and meta-analysis. Br J Sports Med.

[15] Oosterhoff J.H.F., Gravesteijn B.Y., Karhade A.V., Jaarsma R.L., Kerkhoffs G., Ring D. (2022). Feasibility of machine learning and logistic regression algorithms to predict outcome in orthopaedic trauma surgery. J Bone Joint Surg Am.

[16] Rossi L.A., Frank R.M., Wilke D., Provencher C.M.T., Millet P.J., Romeo A. (2021). Evaluation and management of glenohumeral instability with associated bone loss: an expert consensus statement using the modified Delphi technique. Arthroscopy.

[17] Sterne J.A.C., White I.R., Carlin J.B., Spratt M., Royston P., Kenward M.G. (2009). Multiple imputation for missing data in epidemiological and clinical research: potential and pitfalls. BMJ.

[18] Steyerberg E.W. (2019).

[19] Tokish J.M., Kuhn J.E., Ayers G.D., Arciero R.A., Burks R.T., Dines D.M. (2020). Decision making in treatment after a first-time anterior glenohumeral dislocation: a Delphi approach by the Neer Circle of the American Shoulder and Elbow Surgeons. J Shoulder Elbow Surg.

[20] Trasolini N.A., Dandu N., Azua E.N., Garrigues G.E., Verma N.N., Yanke A.B. (2021). Inconsistencies in controlling for risk factors for recurrent shoulder instability after primary arthroscopic Bankart repair: a systematic review. Am J Sports Med.

[21] Verweij L.P.E., van Spanning S.H., Grillo A., Kerkhoffs G., Priester-Vink S., van Deurzen D.F.P. (2021). Age, participation in competitive sports, bony lesions, ALPSA lesions, > 1 preoperative dislocations, surgical delay and ISIS score > 3 are risk factors for recurrence following arthroscopic Bankart repair: a systematic review and meta-analysis of 4584 shoulders. Knee Surg Sports Traumatol Arthrosc.

[22] Williamson P.R., Altman D.G., Bagley H., Barnes K.L., Blazeby J.M., Brookes S.T. (2017). The COMET handbook: version 1.0. Trials.

[23] Yang D.X., Khera R., Miccio J.A., Jairam V., Chang E., Yu J.B. (2021). Prevalence of missing data in the national cancer database and association with overall survival. JAMA Netw Open.

