# Supplementary material for: High variability in what is considered important to report following instability surgery: a Delphi study among Dutch shoulder specialists
Source: JSES Int. 2023 Jul 23;7(6):2316–20. doi: 10.1016/j.jseint.2023.06.020 (PMC10638571; doi:10.1016/j.jseint.2023.06.020)
Supplement: Supplements 1 [file mmc1.docx]

**Supplement 1.** Each element is presented here with the percentage of the panel that gave the element a score of 7 or higher during round two (light blue) and three (dark blue).

**Legenda:**


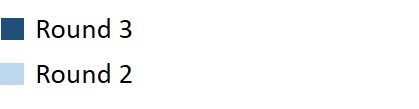


**Arthroscopic Bankart repair: standard and patient factors**

**Arthroscopic Bankart repair: preoperative tests**

**Arthroscopic Bankart repair: labrum**

**Arthroscopic Bankart repair: lesions**

**Arthroscopic Bankart repair: surgery specific/technical elements**

**Arthroscopic Bankart repair: fixation**

**Arthroscopic Bankart repair: additional surgical interventions**

**Arthroscopic Bankart repair: rehabilitation**

**Open Latarjet procedure: standard and patient factors**

**Open Latarjet procedure: preoperative tests**

**Open Latarjet procedure: lesions**

**Open Latarjet procedure: graft**

**Open Latarjet procedure: fixation**

**Open Latarjet procedure: surgery specific/technical elements**

**Open Latarjet procedure: rehabilitation**
